# Association of Teaching Status and Mortality After Cancer Surgery

**DOI:** 10.1097/AS9.0000000000000073

**Published:** 2021-07-23

**Authors:** Miranda B. Lam, Kristen E. Riley, Winta Mehtsun, Jessica Phelan, E. John Orav, Ashish K. Jha, Laura G. Burke

**Affiliations:** From the * Department of Health Policy and Management, Harvard School of Public Health, Boston, MA; † Department of Radiation Oncology, Brigham and Women’s Hospital/Dana Farber Cancer Institute, Boston, MA; ‡ Harvard Medical School, Boston, MA; § Department of Surgery, Brigham and Women’s Hospital/Dana Farber Cancer Institute, Boston, MA; ∥ Division of General Internal Medicine, Department of Medicine, Brigham and Women’s Hospital, Boston, MA; ¶ Department of Biostatistics, Harvard T.H. Chan School of Public Health, Boston, MA; # Brown University School of Public Health, Providence, RI; ** Department of Emergency Medicine, Beth Israel Deaconess Medical Center, Boston, MA.

## Abstract

To examine patient outcomes for 9 cancer-specific procedures performed in teaching versus nonteaching hospitals.

**Background:**

Few contemporary studies have evaluated patient outcomes in teaching versus nonteaching hospitals across a comprehensive set of cancer-specific procedures.

**Methods:**

Use of national Medicare data to compare 30-, 60-, and 90-day mortality rates in teaching and nonteaching hospitals for cancer-specific procedures. Risk-adjusted 30-day, all-cause, postoperative mortality overall and for each specific surgery, as well as overall 60- and 90-day mortality rates, were assessed.

**Results:**

The sample consisted of 159,421 total cancer surgeries at 3151 hospitals. Overall 30-day mortality rates, adjusted for procedure type, state, and invasiveness of procedure were 1.3% lower at major teaching hospitals (95% confidence interval [CI], −1.6% to −1.1%; *P* < 0.001) relative to nonteaching hospitals. After accounting for patient characteristics, major teaching hospitals continued to demonstrate lower mortality rates compared with nonteaching hospitals (−1.0% difference [95% CI, −1.2% to −0.7%]; *P* < 0.001). Further adjustment for surgical volume as a mediator reduced the difference to −0.7% (95% CI, −0.9% to −0.4%; *P* < 0.001). Cancer surgeries for 4 of the 9 disease sites (bladder, lung, colorectal, and ovarian) followed this overall trend. Sixty- and 90-day overall mortality rates, adjusted for procedure type, state, and invasiveness of procedure showed that major teaching hospitals had a 1.7% (95% CI, −2.1% to −1.4%; *P* < 0.001) and 2.0% (95% CI, −2.4% to −1.6%; *P* < 0.001) lower mortality relative to nonteaching hospitals. These trends persisted after adjusting for patient characteristics.

**Conclusions:**

Among cancer-specific procedures for Medicare beneficiaries, major teaching hospital status was associated with lower 30-, 60-, and 90-day mortality rates overall and across 4 of the 9 cancer types.

## INTRODUCTION

Compared with other conditions and procedures, cancer surgery and care is oftentimes more complex and costly and becoming even more so with the emergence of new technologies and treatments.^1,2^ Given this complexity, patient outcomes for cancer procedures vary substantially across hospital types, with up to a fourfold difference in mortality between hospitals.^3^ This variation in outcomes, along with the established relationship between surgical volume and mortality for cancer procedures,^4,5^ has inspired efforts to improve quality by centralizing cancer surgery.^6,7^ Patient safety groups and health systems have begun to support minimum-volume standards for cancer surgeries, encouraging patients to prioritize higher-volume hospitals when seeking cancer care.^8,9^

These efforts have resulted in an increasing number of complex cancer procedures performed in academic medical centers,^10^ which tend to be higher volume than community-based hospitals. While teaching hospitals have demonstrated improved outcomes for some surgical procedures compared with nonteaching hospitals,^11,12^ it is still unclear whether these trends persist for cancer patients, specifically, and across various types of cancer procedures. Considering that teaching hospitals tend to be considered more expensive than community hospitals,^13,14^ and that cancer care already poses a significant financial burden to patients and payers,^15–17^ it is important to understand whether patient outcomes differ between teaching and nonteaching hospitals. Further, given that the centralization of cancer surgery away from the community to major teaching hospitals may increase disparities in access to care,^18,19^ it is necessary to establish whether these settings truly demonstrate improved outcomes across a comprehensive set of cancer surgeries in a contemporary cohort.

Therefore, in this study, we sought to answer 3 key questions using Medicare data. First, to what degree does 30-day overall mortality for cancer surgery differ in teaching hospitals compared with nonteaching hospitals? Second, to what extent does 30-day mortality among teaching and nonteaching hospitals vary by type of cancer surgery? Third, do these differences, if any, persist at 60- and 90-days post-surgery? (Supplemental Appendix, http://links.lww.com/AOSO/A40).

## METHODS

### Data Sources

We linked several data sources for this study. The Medicare Inpatient Claims data from 2016 to 2017 was linked to the Beneficiary Denominator File and Medicare Enrollment Database, which provided patient-level variables, including basic demographic characteristics, primary causes and dates of hospitalization, and comorbidities, mortality, and procedures. Medicaid eligibility was determined using the State Buy-In Coverage Count variable. Any beneficiary with at least 1 month of state buy-in (Medicare premium paid by the state) was considered Medicaid eligible. We used the American Hospital Association (AHA) annual survey and excluded federal hospitals and specialty hospitals other than cancer hospitals (ie, psychiatric hospitals and children’s hospitals). Hospital admissions for cancer surgery without corresponding data in the AHA annual survey (0.7% of total) were excluded as it was not possible to determine the primary predictor of interest, teaching status, for these admissions. The Institutional Review Board at the Harvard T.H. Chan School of Public Health approved this study.

### Primary Exposure: Hospital Teaching Status

The primary exposure of interest was hospital teaching status. Consistent with prior research,^11,20–22^ all hospitals were placed into 1 of 3 categories based on their response to the AHA survey: major teaching hospitals (those that are members of the Council of Teaching Hospitals [COTH]), minor teaching hospitals (non-COTH members that had a medical school affiliation reported to the American Medical Association), and nonteaching hospitals (all other institutions). Both major and minor teaching hospitals have residents. By including both major and minor teaching hospitals, which differ based on COTH membership as well as by intern and residents to bed ratio, we were able to examine whether the differences in outcomes established for other conditions persist for cancer patients specifically.^11,21,23^ For each hospital, data were obtained on its teaching status, size, geographic region, ownership (for-profit, private nonprofit, or public), urban versus rural location, and presence or absence of a medical and cardiac intensive care unit.

### Patients

Using claims data for the period from January 1, 2016, to September 30, 2017, from the 100% Medicare inpatient files, we identified fee-for-service beneficiaries enrolled in Medicare Part A who had a major surgical resection for colorectal, bladder, esophageal, kidney, liver, ovarian, pancreatic, lung, or prostate cancer. We used diagnosis and procedure codes from the *International Classification of Disease, Tenth Revision, Clinical Modification* (Supplemental Table 1, http://links.lww.com/AOSO/A40). Surgeries included had a claim in both the diagnosis code and a procedure code for the same type of cancer. We excluded patients who had undergone 2 or more different oncologic procedures on the same day. We categorized the procedure codes by type and extent of surgery, as well as by whether the procedures were endoscopic, percutaneous, both, or neither. While there is mixed evidence on the volume-outcomes relationship for less invasive procedures,^24–26^ we opted to include a comprehensive set of procedures in our analysis given the increasing use of less invasive surgical techniques^27^ as well as the relationship between outcomes and hospital characteristics established in many studies. For each hospitalization for cancer surgery, the patient’s age, sex, race, Medicaid eligibility, and Elixhauser chronic conditions were obtained.

### Outcomes

The primary outcome of interest was risk-adjusted 30-day, all-cause, postoperative mortality (death within 30 days of the operation).^28^ Thirty-day mortality rates were calculated for all cancer surgeries and then by each cancer surgery separately. These cancer surgeries were chosen as they are common, complex cancer surgeries and have been used in prior studies.^29^ For secondary outcomes, 60- and 90-day mortality rates for the combined cancer surgery group were also calculated. This was performed to examine if any differences in early mortality persisted over time.

### Statistical Analysis

For all cancer surgeries, we first compared patient and hospital characteristics by teaching hospital status. Kaplan-Meier survival curves for major, minor, and nonteaching hospitals were created, with censoring of patients still alive at 90 days. We then used a multivariable patient-level linear probability regression model to examine the relationship between teaching hospital status and postoperative mortality. We chose a linear probability model to preserve the interpretability of linear trends in mortality rates. We used a generalized estimating equation approach to adjust for correlated outcomes within hospitals. Our initial model incorporated fixed effects for the state in which the hospital was located, to account for geographic differences in the healthcare delivery environment as well as for by procedure group and invasiveness of the procedure (endoscopic, percutaneous, both, or neither) (Supplemental Table 1, http://links.lww.com/AOSO/A40). To account for differences in patient severity by hospital teaching status, our main model further incorporated beneficiary age, sex, dual status, Elixhauser comorbidities, and Medicaid eligibility. This allowed us to calculate overall and by cancer type, risk-adjusted estimates of mortality for patients who had surgery at teaching hospitals versus nonteaching hospitals. The initial and main models were rerun with 60- and 90-day mortality as the outcomes.

### Sensitivity Analysis: Mortality for Nonopen Cancer Surgery

It is possible that nonopen, less invasive cancer surgeries may not benefit from being performed at teaching hospitals as more complex, open procedures. Thus, we reran 30-day mortality analyses for patients who underwent nonopen surgeries.

### Sensitivity Analysis: Surgical Volume as a Mediator

We examined surgical volume as a potential mediator of any differences in outcomes by hospital teaching status. Given the varied incidence of each cancer type, we divided hospitals volume into tertiles for each of the 9 cancer types.^4,30–33^ We then reran the final adjusted model including surgical volume as a mediator, as well as stratifying by surgical volume.^34^

## RESULTS

### Hospital and Patient Characteristics

The analytic sample consisted of 159,421 total cancer surgeries at 3151 hospitals (Table 1). Of the 3,151 hospitals, 238 (7.6%) were major teaching hospitals and accounted for 33.3% of the cancer surgeries in the sample, 1033 (32.8%) were minor teaching hospitals and accounted for 37.5% of cancer surgeries, and 1880 (60.0%) were nonteaching hospitals and accounted for 29.2% of cancer surgeries. Patient characteristics for cancer surgeries among major teaching, minor teaching, and nonteaching hospitals are presented in Table 1, as are key characteristics of the hospitals within each group.

**TABLE 1.** Comparison of Baseline Hospital and Patient Characteristics for Cancer Surgeries by Teaching Status* (2016–2017)

	Major Teaching	Minor Teaching	Nonteaching
Hospital characteristics			
Number of hospitals	238	1033	1880
Size			
Small (<99 beds)	1 (0.4)	171 (16.6)	926 (49.3)
Medium (100–399 beds)	48 (20.2)	661 (64.0)	893 (47.5)
Large (>400 beds)	189 (79.4)	201 (19.5)	61 (3.2)
Region			
Northeast	72 (30.3)	197 (19.1)	223 (11.9)
Midwest	55 (23.1)	260 (25.2)	548 (29.1)
South	75 (31.5)	338 (32.7)	749 (39.8)
West	36 (15.1)	238 (23.0)	360 (19.1)
Profit status			
For-profit	5 (2.1)	174 (16.8)	409 (21.8)
Nonprofit	183 (76.9)	745 (72.1)	1181 (62.8)
Government, nonfederal	50 (21.0)	114 (11.0)	290 (15.4)
Urban location	238 (100)	984 (95.3)	1594 (84.8)
CoC approved	211 (88.7)	570 (55.2)	544 (28.9)
Mean hospital IRB ratio	0.43	0.10	0.01
Patient characteristics			
Number of cancer surgeries	53,027	59,802	46,592
Age, mean	74.1	75.2	75.8
Female	39.4	40.7	40.0
Ethnicity/race			
White	85.0	87.2	88.4
Black	8.2	7.1	6.5
Hispanic	1.0	0.9	1.1
Other	5.9	4.7	3.9
Medicaid eligible	10.3	10.7	12.2
Comorbidities†			
Congestive heart failure	6.9	8.8	9.6
Valvular disease	6.0	6.0	6.0
Pulmonary circulation disease	0.9	0.9	0.9
Peripheral vascular disease	6.5	6.7	6.8
Paralysis	1.1	1.4	1.5
Other neurological disorders	5.2	5.8	6.3
Chronic pulmonary disease	20.9	23.1	24.2
Diabetes	24.9	25.7	26.5
Hypothyroidism	13.9	13.8	13.8
Renal failure	11.9	12.7	13.6
Liver disease	5.4	3.1	2.7
Peptic ulcer × bleeding	0.7	0.9	1.0
AIDS	0.1	0.0	0.0
Rheumatoid arthritis	2.7	2.6	2.4
Coagulopathy	4.7	4.0	4.0
Obesity	13.8	12.3	12.1
Weight loss	7.8	8.6	8.9
Fluid and electrolyte disorders	19.8	22.9	25.1
Chronic blood loss anemia	1.6	3.6	4.6
Deficiency anemias	13.8	18.9	21.6
Psychoses	0.8	1.1	1.0
Depression	8.6	8.3	7.7
Hypertension	65.1	67.4	68.3

*Major teaching hospitals were members of Council of Teaching Hospitals (COTH). Minor teaching hospitals had a medical school but no COTH affiliation. Nonteaching hospitals had neither COTH membership nor medical school affiliation.

†Comorbidities were derived from Elixhauser conditions.

CoC indicates Commission on Cancer; IRB, intern resident bed.

### Thirty-Day Mortality: Total Cohort

**FIGURE 1.**
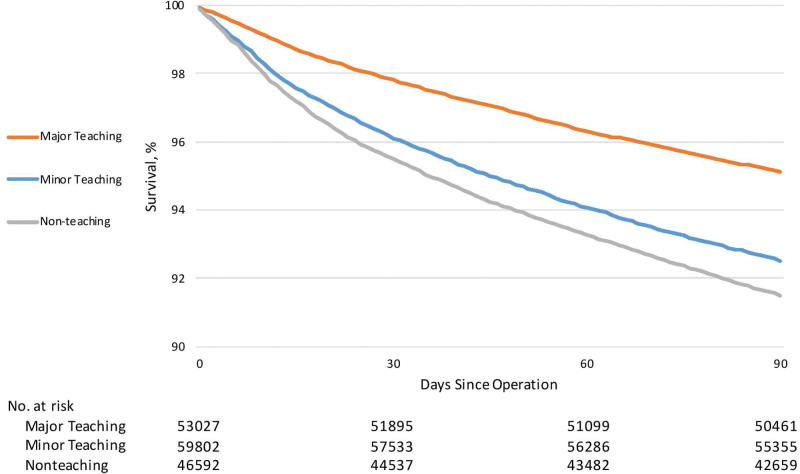
Kaplan Meier survival curves after cancer surgery.

Kaplan-Meier survival curves, presented in Figure 1, show cumulative incidence of mortality at major teaching, minor teaching, and nonteaching hospitals out to 90 days. The 30-day mortality rates adjusted for procedure, state, and invasiveness of the procedure (endoscopic, percutaneous, both, neither) for major teaching, minor teaching, and nonteaching hospitals were 2.7%, 3.8%, and 4.0%, respectively, with major teaching hospitals having 1.3% lower mortality (95% confidence interval [CI], −1.6% to −1.1%; *P* < 0.001) relative to nonteaching hospitals (Table 2). This pattern persisted after additionally accounting for patient characteristics (2.9% mortality at major vs 3.7% at minor vs 3.9% at nonteaching; −1.0% difference [95% CI, −1.2% to −0.7%] between major teaching and nonteaching hospitals; *P* < 0.001). Similar results were found among less invasive, nonopen cancer surgeries (Supplemental Table 3, http://links.lww.com/AOSO/A40).

**TABLE 2.** Comparison of 30-Day Mortality for Cancer Surgeries by Hospital Teaching Status* (2016–2017)

	Hospital Teaching Status			Difference (95% CI), *P*		
	Major Teaching	Minor Teaching	Nonteaching	Major vs Nonteaching†	Major vs Minor Teaching‡	Minor vs Nonteaching§
Surgeries, no.	53,027	59,802	46,592			
Mortality Adjusted for procedure, state, and endo/perc	2.7%	3.8%	4.0%	−1.3% (−1.6% to −1.1%) *P* < 0.001	−1.1% (−1.4% to −0.9%) *P* < 0.001	−0.2% (−0.5% to 0.0%) *P* = 0.102
Mortality Adjusted for procedure, state, endo/perc, and patient characteristics∥	2.9%	3.7%	3.9%	−1.0% (−1.2% to −0.7%) *P* < 0.001	−0.8% (−1.0% to −0.6%) *P* < 0.001	−0.2% (−0.4% to 0.1%) *P* = 0.229

*Major teaching hospitals were members of COTH. Minor teaching hospitals had a medical school but no COTH affiliation. Nonteaching hospitals had neither COTH membership nor medical school affiliation.

†Difference between mean value and *P* value for major teaching subtracted from the mean value for nonteaching.

‡Difference between mean value and *P* value for major teaching subtracted from the mean value for minor teaching.

§Difference between mean value and *P* value for minor teaching subtracted from the mean value for nonteaching.

∥Patient Characteristics include age, sex, Medicaid eligibility, Elixhauser conditions.

### Thirty-Day Mortality: By Cancer Surgery

**FIGURE 2.**
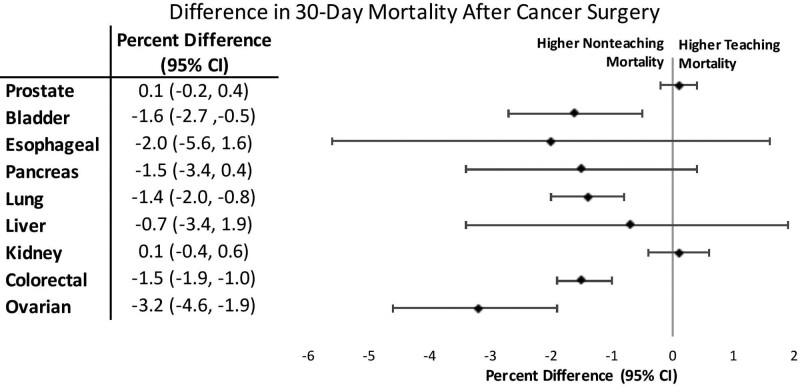
Forest plot of difference in 30-day mortality (by cancer surgery and overall) between major teaching and nonteaching hospitals.

The adjusted analyses of overall 30-day mortality stratified by cancer type for major teaching, and nonteaching hospitals are presented in Figure 2 and Table 3. The significant association between major teaching hospital status and lower 30-day mortality was again observed for 4 of the 9 disease sites (bladder, lung, colorectal, and ovarian). The lower mortality at major teaching hospitals relative to nonteaching hospitals was not significant for patients undergoing esophageal, pancreas, and liver cancer surgery and there was no difference in mortality between teaching and nonteaching hospitals for prostate and kidney cancer patients.

**TABLE 3.** Comparison of 30-Day Adjusted Mortality* by Cancer Surgery and Hospital Teaching Status† (2016–2017)

	Major Teaching	Minor Teaching	Nonteaching	Difference (95% CI), *P*‡
Prostate	0.76% (n = 9517)	0.77% (n = 10,732)	0.65% (n = 7802)	0.1% (−0.2% to 0.4%) *P* = 0.451
Bladder	5.95% (n = 5224)	6.70% (n = 5384)	7.52% (n = 4368)	−1.6% (−2.7% to −0.5%) *P* = 0.005
Esophageal	5.01% (n = 1127)	7.08% (n = 575)	6.97% (n = 227)	−2.0% (−5.6% to 1.6%) *P* = 0.286
Pancreas	2.84% (n = 3413)	4.16% (n = 1435)	4.34% (n = 581)	−1.5% (−3.4% to 0.4%) *P* = 0.121
Lung	2.09% (n = 10,364)	3.01% (n = 9977)	3.50% (n = 6412)	−1.4% (−2.0% to −0.8%) *P* < 0.001
Liver	3.24% (n = 2001)	4.86% (n = 521)	3.99% (n = 197)	−0.7% (−3.4% to 1.9%) *P* = 0.585
Kidney	1.52% (n = 6786)	1.78% (n = 7093)	1.41% (n = 5044)	0.1% (−0.4% to 0.6%) *P* = 0.662
Colorectal	4.00% (n = 11,772)	5.35% (n = 21,709)	5.47% (n = 20,860)	−1.5% (−1.9% to −1.0%) *P* < 0.001
Ovarian	1.42% (n = 2823)	3.19% (n = 2376)	4.65% (n = 1101)	−3.2% (−4.6% to −1.9%) *P* < 0.001

*Adjusted for procedure, state, endo/perc, and patient characteristics (age, sex, Medicaid eligibility, Elixhauser conditions).

†Major teaching hospitals were members of COTH. Minor teaching hospitals had a medical school but no COTH affiliation. Nonteaching hospitals had neither COTH membership nor medical school affiliation.

‡Difference between mean value and *P* value for major teaching subtracted from the mean value for nonteaching.

### Sixty- and 90-Day Mortality

To determine whether these associations persisted over time, we also analyzed 60- and 90-day mortality for our overall cohort. Overall 60-day mortality adjusted for procedure type, state, and invasiveness of procedure for major teaching, minor teaching, and nonteaching hospitals were 4.4%, 5.8%, and 6.1%, respectively, with major teaching hospitals having 1.7% lower mortality (95% CI, −2.1% to −1.4%; *P* < 0.001) relative to nonteaching hospitals (Table 4). This pattern persisted after additionally accounting for patient characteristics (4.7% mortality at major vs 5.7% at minor vs 5.9% at nonteaching; −1.2% difference [95% CI, −1.5% to −0.9%] between major teaching and nonteaching hospitals; *P* < 0.001).

**TABLE 4.** Sixty- and 90-Day Mortality of Cancer Surgeries by Hospital Teaching Status*

	Hospital Teaching Status			Difference (95% CI), *P*		
	Major Teaching	Minor Teaching	Major Teaching	Major vs Nonteaching†	Major vs Minor Teaching‡	Minor vs Nonteaching§
60-d mortality						
Mortality Adjusted for procedure, state, and endo/perc	4.4%	5.8%	6.1%	−1.7% (−2.1% to −1.4%) *P* < 0.001	−1.5% (−1.8% to −1.1%) *P* < 0.001	−0.2% (−0.6% to 0.1%) *P* = 0.145
Mortality Adjusted for procedure, state, endo/perc, and patient characteristics∥	4.7%	5.7%	5.9%	−1.2% (−1.5% to −0.9%) *P* < 0.001	−1.0% (−1.3% to −0.7%) *P* < 0.001	−0.2% (−0.5% to 0.2%) *P* = 0.309
90-d mortality						
Mortality Adjusted for procedure, state, and endo/perc	5.7%	7.4%	7.7%	−2.0% (−2.4% to −1.6%) *P* < 0.001	−1.7% (−2.1% to −1.3%) *P* < 0.001	−0.3% (−0.7% to 0.1%) *P* = 0.114
Mortality Adjusted for procedure, state, endo/perc, and patient characteristics∥	6.1%	7.2%	7.5%	−1.4% (−1.7% to −1.0%) *P* < 0.001	−1.2% (−1.5% to −0.8%) *P* < 0.001	−0.2% (−0.6% to 0.2%) *P* = 0.256

*Major teaching hospitals were members of COTH. Minor teaching hospitals had a medical school but no COTH affiliation. Nonteaching hospitals had neither COTH membership nor medical school affiliation.

†Difference between mean value and *P* value for major teaching subtracted from the mean value for nonteaching.

‡Difference between mean value and *P* value for major teaching subtracted from the mean value for minor teaching.

§Difference between mean value and *P* value for minor teaching subtracted from the mean value for nonteaching.

∥Adjusted for procedure, state, and patient characteristics (age, sex, Medicaid eligibility, Elixhauser conditions).

Overall 90-day mortality rates adjusted for procedure type, state, and invasiveness of procedure for major teaching, minor teaching, and nonteaching hospitals were 5.4%, 7.4%, and 7.7%, respectively, with major teaching hospitals having 2.0% lower mortality (95% CI, −2.4% to −1.6%; *P* < 0.001) relative to nonteaching hospitals (Table 4). This pattern persisted after additionally accounting for patients characteristics (6.1% mortality at major vs 7.2% at minor vs 7.5% at nonteaching; −1.4% difference [95% CI, −1.7% to −1.0%] between major teaching and nonteaching hospitals; *P* < 0.001).

### Surgical Volume as a Mediator

The 30-day mortality difference between major teaching and nonteaching hospitals was persistent but was lesser in magnitude (−0.7% [95% CI, −0.9% to −0.4%; *P* < 0.001]) when we incorporated surgical volume into the model (Table 5; Supplemental Table 2, http://links.lww.com/AOSO/A40). When broken down by categories of surgical volume, there was a statistically significantly lower 30-day mortality for high volume major teaching versus high-volume nonteaching hospitals (−0.8% difference [95% CI, −1.1 to −0.5]; *P* < 0.001), while the differences by hospital teaching status were not significant for medium- and low-volume hospitals.

**TABLE 5.** Thirty-Day Mortality* by Surgical Volume and Hospital Teaching Status† (2016–2017)

	Major Teaching	Minor Teaching	Nonteaching	Difference (95% CI), *P*‡
30-d mortality* adjusted for surgical volume as a mediator				
Mortality Adjusted for procedure, state, patient characteristics, endo/perc, and surgical volume	3.0%	3.7%	3.7%	−0.7% (−0.9% to −0.4%) *P* < 0.001
30-d mortality* by surgical volume				
Low volume	6.0% (n = 381)	6.3% (n = 2809)	6.2% (n = 5026)	−0.2% (−2.2% to 1.8%) *P* = 0.82
Medium volume	4.4% (n = 2397)	5.0% (n = 11,501)	4.7% (n = 14,723)	−0.3% (−1.2% to 0.7%) *P* = 0.57
High volume	2.6% (n = 50,249)	3.2% (n = 45,492)	3.4% (n = 26,843)	−0.8% (−1.1% to −0.5%) *P* < 0.001

*Adjusted for procedure, state, patient characteristics (age, sex, Medicaid eligibility, Elixhauser conditions).

†Major teaching hospitals were members of COTH. Minor teaching hospitals had a medical school but no COTH affiliation. Nonteaching hospitals had neither COTH membership nor medical school affiliation.

‡Difference between mean value and *P* value for major teaching subtracted from the mean value for nonteaching.

## DISCUSSION

In an analysis of 159,421 Medicare beneficiaries undergoing major surgical resection for colorectal, bladder, esophageal, kidney, liver, ovarian, pancreatic, lung, or prostate cancer from January 2016 to September 2017, major teaching hospital status was associated with lower 30-day postoperative mortality relative to procedures performed at nonteaching hospitals. The finding of lower mortality at major teaching hospitals were observed across 7 of the 9 cancer procedures in this study. While only 4 of the procedures reached statistical significance, the effects were of similar magnitude for the other procedures. Furthermore, these mortality differences by hospital teaching status persisted over time, with differences apparent at 60 and 90 days.

Our study findings coincide with 2 concurrent, and at times conflicting, trends in cancer care. First, the movement to steer patients toward high-volume tertiary centers for cancer-specific procedures^8^ is motivated by a large body of evidence showing surgical volume is associated with better patient outcomes.^3,4,12,35,36^ However, despite such efforts, a large proportion of patients pursue complex cancer surgery outside of tertiary care centers. In fact, in our study, we found about 30% of patients had cancer surgery at a nonteaching hospital. The slow adoption of the regionalization of cancer surgery in the United State is multifactorial but could include travel barriers, financial impediments, and patient reluctance to access care outside of their home communities.^37,38^ Our study found that having cancer surgery at a major teaching hospital for these common cancers was associated with better outcomes, suggesting that efforts to centralize care at these centers may be justified. Policy efforts focused on regionalization of cancer surgery must mitigate the barriers which prevent many patients from accessing care at tertiary academic centers, such as travel, lack of support structures outside of a patient’s local community, and financial burden. Furthermore, when considering the larger policy environment, there has been a concomitant effort to narrow healthcare networks to reduce overall costs. There is some evidence that this has been impacting cancer care^39,40^ and can limit access to major teaching hospitals in an effort to control costs. Our findings suggest that for cancer care, exclusion of major teaching hospitals has the potential to worsen patient outcomes.

The mortality differences between major teaching hospitals and nonteaching hospitals demonstrated in this study suggest that a shift away from major teaching hospitals may have substantial consequences for patients. The 1% 30-day mortality difference between major teaching and nonteaching hospitals, for example, equates to a number needed to treat of 100, meaning that for every 100 patients undergoing cancer surgery, 1 death could have been prevented if they received surgery at a teaching hospital instead of a nonteaching hospital. When considering the 47,000 Medicare beneficiaries included in this study, 470 deaths could have been prevented by providing surgery in a major teaching hospital, assuming the differences represent a causal relationship. The number of preventable deaths are even greater for 60- and 90-day mortality (566 and 662, respectively). The magnitude of the association between hospital teaching status and outcomes compares favorably to other well-established relationships in the surgical health services literature.^35,41^ Given this tangible impact on human lives, it is important to consider these mortality differences when making policy decisions related to cancer care.

There are several possible reasons for the observed differences in mortality by hospital teaching status. First, major teaching hospitals in our sample tended to be larger than nonteaching hospitals, with 79.4% of major teaching hospitals categorized as large (>400 beds) and only 3.2% of nonteaching hospitals. Our results suggest that higher surgical volume accounted for some, but not all, of the association between hospital teaching status and surgical outcomes for cancer care as the lower mortality seen at major teaching hospitals was observed even when examining hospitals with the highest surgical volume. Teaching hospitals may provide greater access to specialty-trained surgeons, which in turn has shown to be beneficial for patient outcomes across a variety of procedures,^42^ including lower mortality for a number of cancer procedures.^43–45^ Furthermore, the better patient outcomes in teaching hospitals identified in our study may be partially attributable to other system-level factors, improved postoperative care by multidisciplinary teams, adoption of new technologies, and availability of complex resources across a spectrum of providers. Prior research has demonstrated a relationship between teaching intensity and availability of patient services and advanced technologies, such as advanced imaging modalities.^46,47^ It is possible that greater technological advances and resources are driving some of the improved patient outcomes observed at teaching hospitals in our study. Additionally, greater access to other nonsurgical specialists at teaching hospitals, such as intensive care specialists, may provide additional safeguards in the face of complications or unexpected clinical deterioration.

Our study adds to a growing body of literature suggesting that treatment at a major teaching hospital is associated with better short- and long-term outcomes for medical and surgical care.^11,46,48,49^ There are a number of studies suggesting a benefit of care at major teaching hospitals for specific cancer types such as leukemia, rectal cancer, and pancreatic cancer.^10,50–53^ Further, of the existing studies examining differences in outcomes for cancer procedures at teaching and nonteaching hospitals, many focus on in-hospital mortality rates and length of stay.^10,48,50,54^ Our study extends on this previous study in a number of ways. First, it evaluates longer-term outcomes by examining 30-, 60-, and 90-day postoperative mortality. Second, it evaluates outcomes across a comprehensive set of cancer procedures to explore whether these differences in persist across disease types. Finally, our study builds on studies from over many years ago that explore the relationship between teaching status and cancer surgery outcomes by examining this relationship in a contemporary cohort to understand whether the recent emphasis on improving surgical outcomes across hospitals has influenced these patterns.

## LIMITATIONS

This study has several limitations. First, this study examined mortality rates for Medicare fee-for-service patients undergoing cancer surgeries, and therefore it is not possible to determine whether these findings would be generalizable to younger populations. Second, we only examined mortality as an indicator of the quality of hospital care and it is possible that other outcomes, such as functional status, may have shown a different relationship with hospital teaching status. Third, only 4 of the procedures reached statistical significance, even though the effects were of similar magnitude for the other procedures, suggesting that the nonsignificance may be related more to smaller sample sizes and decreased power than a true lack of an effect. It will be important for studies such as these to be re-evaluated over time and with different datasets. Finally, our study is observational and it is possible that the severity of patient differences in outcomes could be related to unmeasured confounders. This study utilized claims data and therefore lacks more granular clinical data such as stage of cancer or date of initial diagnosis. While we adjusted for patient characteristics such as age, sex, Medicaid eligibility, Elixhauser conditions, and procedure complexity, there may be other patient-level factors unavailable in the Medicare claims database that differ between teaching and nonteaching hospitals that are influencing our results. However, in order for such confounding to explain our study findings, this would require patients referred for cancer care at major teaching hospitals to be systematically healthier than those treated at nonteaching settings, which seems unlikely given the overall tendency to refer higher-risk patients for tertiary care.^55^

## CONCLUSIONS

Among patients undergoing common, complex cancer surgeries for US Medicare beneficiaries, major teaching hospital status was associated with lower mortality rates compared with nonteaching hospitals.
